# Tumor Macroenvironment and Metabolism

**DOI:** 10.1053/j.seminoncol.2014.02.005

**Published:** 2014-04

**Authors:** Wael Al-Zhoughbi, Jianfeng Huang, Ganapathy S. Paramasivan, Holger Till, Martin Pichler, Barbara Guertl-Lackner, Gerald Hoefler

**Affiliations:** a Institute of Pathology, Medical University of Graz, Graz, Austria; b Department of Paediatric and Adolescent Surgery, Medical University of Graz, Graz, Austria; c Department of Internal Medicine, Medical University of Graz, Graz, Austria

## Abstract

In this review we introduce the concept of the tumor macroenvironment and explore it in the context of metabolism. Tumor cells interact with the tumor microenvironment including immune cells. Blood and lymph vessels are the critical components that deliver nutrients to the tumor and also connect the tumor to the macroenvironment. Several factors are then released from the tumor itself but potentially also from the tumor microenvironment, influencing the metabolism of distant tissues and organs. Amino acids, and distinct lipid and lipoprotein species can be essential for further tumor growth. The role of glucose in tumor metabolism has been studied extensively. Cancer-associated cachexia is the most important tumor-associated systemic syndrome and not only affects the quality of life of patients with various malignancies but is estimated to be the cause of death in 15%–20% of all cancer patients. On the other hand, systemic metabolic diseases such as obesity and diabetes are known to influence tumor development. Furthermore, the clinical implications of the tumor macroenvironment are explored in the context of the patient’s outcome with special consideration for pediatric tumors. Finally, ways to target the tumor macroenvironment that will provide new approaches for therapeutic concepts are described.

Complications of a malignant tumor can be either (1) local due to direct effects of the primary tumor or metastatic lesions on the surrounding tissues, or (2) systemic. Tumors may cause systemic effects by releasing soluble factors into blood or lymph vessels^1^ or via immune reactions caused by cross-reactivity between cancer cells and normal tissues.^2^ Some of these systemic complications can be categorized under the well-known paraneoplastic syndromes.^2^ Perhaps the most common effect tumors exert on their macroenvironment is cancer-associated cachexia. Other systemic changes, though pathological, are subclinical and might not only be beneficial as clinical markers for prognosis and therapy prediction^3^ but also may help to understand the mechanisms causing systematic complications.

With recent advances in cancer therapy, patients live longer and, therefore, it is of utmost importance to improve the quality of life during this time. In this context, addressing systemic complications as a target for intensive research and development of treatment options is imperative. This review aims to introduce the concept of tumor macroenvironment, explore it in the context of the tumor microenvironment, and discuss the clinical and therapeutic implications of this concept.

## Tumor Microenvironment

Before discussing a definition of the tumor macroenvironment, we will briefly explore the cellular elements of the tumor microenvironment and consider their local and systemic interactions.

### Tumor-Associated Inflammation and Angiogenesis

As early as 1863 Rudolf Virchow observed that tumor tissues are infiltrated by immune cells; he was also the first to hypothesize a direct link between inflammation and cancer.^4^ This hypothesis is now widely accepted and a large body of research supports this fact. About 15% of human cancers are estimated to arise from sites of infection or chronic inflammation.^5^ Moreover, the majority of solid tumors exhibit infiltration by immune cells and release pathological levels of cytokines into the surrounding tissue and/or into the bloodstream.

The local effect of cytokines released into the tumor microenvironment has been reviewed extensively.^6^ The interaction between these cytokines and the tumor microenvironment affects tumor growth and remodeling of the tumor microenvironment. Critical components of the tumor microenvironment are newly synthesized blood and lymph vessels, which represent key events in tumor growth that are driven by the metabolic needs of proliferating cells, including oxygen and nutrients, and are mediated by pro-inflammatory cytokines. A key event that initiates or enhances the angiogenic process is stabilization of hypoxia inducible factor 1-alpha (HIF1α) in the hypoxic tumor microenvironment.^7^ Interleukin-1 beta (IL-1β) is an important mediator of tumor angiogenesis.^8^ Together with prostaglandin E2 (PGE2), IL-1β upregulates HIF1α protein levels and activates vascular endothelial growth factor (VEGF), a reaction that is mainly mediated by the nuclear factor κB (NFκB) pathway.^9^ This cascade of gene activation illustrates one important example of a mechanistic explanation for the role of inflammation in tumor development. Other mechanisms supporting angiogenesis have been reviewed elsewhere.^10^ The newly synthesized blood and lymph vessels not only contribute to delivery of oxygen and nutrients to tumor cells thereby supporting tumor growth^10^ but also allow tumor cells to release a wide range of soluble factors into the bloodstream. Mechanistically, this represents the key event connecting the tumor microenvironment with the whole body of the patient exerting systemic biological effects. We suggest using the term “tumor macroenvironment” to define the pathological interaction between the tumor cells, as well as the tumor microenvironment with other organs and systems of the body.

## Tumor Macroenvironment Versus Tumor Microenvironment

Unlike in normal tissue, cellular proliferation in tumors is an uncontrolled process. During the early stages of tumorigenesis, two main signaling types dominate in the tumor microenvironment to support tumor cell proliferation. The first type of signaling increasing proliferation constitutes autocrine stimulation among tumor cells themselves. Tumor cells may release growth factor ligands that bind to receptors on the surface of tumor cells, thereby stimulating proliferation.^11^ The second type of signaling constitutes paracrine interaction between tumor cells and other components of the microenvironment. Factors released from tumor cells can stimulate normal cells to produce growth factors to which tumor cells respond subsequently.^12^ When the size of the tumor reaches the oxygen and nutrient diffusion limit, tumor cells encounter not only a profound metabolic challenge but also hypoxia and nutrient deprivation.^13^

**Figure 1:**
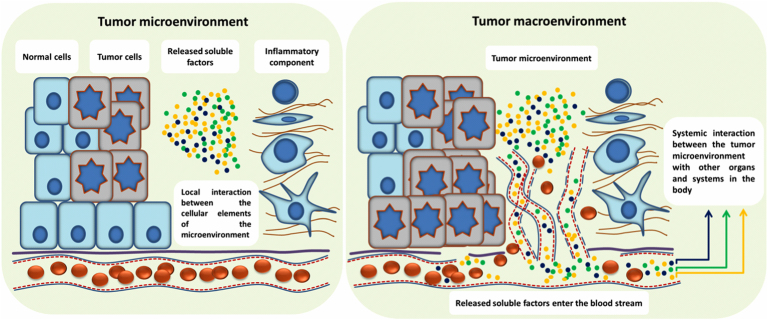
The tumor macroenvironment concept. A simplified schematic of the tumor micro- and macroenvironment: tumor development is a multi-step process that may take place over several years. Transition from normal cell(s) to genetically abnormal cell(s) occurs at the beginning. This transition is a relatively slow process and often clinically silent. When transformed cells start dividing and invade the neighboring tissues, the tumor microenvironment evolves. At this step, cancer cells may face destructive effects of the innate and adaptive immune systems. However, selected cancer cells are able to escape the antitumor immune response, resume growth, proliferate and shape their microenvironment. Importantly, the reciprocal—but abnormal—interactions between cancer cells and the surrounding tissue are mainly localized and limited to the microenvironment at this stage (left). If cancer cells remain undetected and untreated, cancer progresses to advanced stages. As a consequence of (1) abnormal localized interaction and (2) uncontrolled cancer cell proliferation and resulting necrosis, several soluble factors are released from the tumor microenvironment. They may function as proangiogenic factors that stimulate recruitment of endothelial progenitor cells to the tumor microenvironment and induce angiogenesis. Tumor-induced angiogenesis is a critical process in tumor development, as it not only supplies the tumor with required nutrients but also allows soluble factors released by the tumor and the microenvironment to enter the blood and/or lymph stream. This leads to an increased complexity of systemic interactions between the tumor and other organs and systems in the body. In contrast to the tumor microenvironment, where the localized auto and paracrine types of interaction are dominating, the systemic pathological interactions constitute the fundamental mechanism of the tumor macroenvironment concept in cancer biology (right).

To survive in this hostile environment, tumor cells deregulate their intrinsic metabolic machinery and, via paracrine signaling, remodel the tumor microenvironment to activate tumor-associated angiogenesis. Though tumor cells are master regulators of the tumor microenvironment, each type of cell in this environment may interact with other neighboring cells.^14^ Soluble factors released, such as chemokines, cytokines, and growth factors, (1) recruit inflammatory cells, fibroblasts, and myeloid cells; (2) reshape the extracellular matrix; and (3) initiate and support neo-vascularization. On the one hand, tumor-induced angiogenesis supports tumor growth, but on the other hand the newly formed blood vessels are tortuous and leaky. This, again, results in a hostile microenvironment that may induce even more aggressive properties of cancer cells. The imperfectly formed network of newly formed blood vessels in close proximity to tumor cells and inflammatory cells results in accumulation and/or release of soluble factors from the tumor microenvironment into the circulation at high levels. This leads to pathological endocrine effects and interaction between the tumor microenvironment and the patient’s organs and systems, resulting in the development of cancer-associated systemic syndromes in the tumor macroenvironment (Figure 1).

## Metabolism of The Tumor Macroenvironment

### Protein and Amino Acid Metabolism

Increased whole-body protein turnover is often associated with tumor growth. This has been well documented in cachectic^15^ and non-cachectic cancer patients.^16^ The decrease in protein synthesis^17^ and the increase in muscle protein degradation in cancer patients^18^ imply that tumors are able to mobilize muscle proteins. Indeed, several studies demonstrated a direct relationship between tumor growth and host protein metabolism. The concept of tumors as “nitrogen traps” was described as early as in 1951 by Mider.^19^ Nitrogen mobilized from tissues represents a potential source of building blocks for rapidly growing tumors.^20^ Tumor growth in fasted rats was similar to that in fed animals, while their body and liver weights were reduced.^21^ Radioactivity from ^14^C-glycine decreased in normal tissues during the rapid growth phase of Flexner-Jobling carcinoma. Conversely, total radioactivity in tumors increased both in fasted and fed animals, indicating that tumors do not lose protein content during starvation, forming a “one-way passage”.^21^

On the cellular level, protein and amino acid metabolism is also deregulated in cancer cells. In contrast to decreased protein synthesis in muscle cells, tumor cells exhibit increased protein synthesis.^22^ mTORC1 is one of the key players involved in phosphorylation of the translational regulators 4E-binding protein 1 and S6 kinase 1.^22^ One of the key changes in cancer cell metabolism is known as “glutamine addiction” since many types of cancer cells require exogenous supply of this non-essential amino acid. The importance of non-essential amino acids in tumor metabolism surpasses glutamine addiction since several recent studies have highlighted the importance of serine and glycine pathways in tumorigenesis.^23^, ^24^ Because these findings are derived from in vitro experiments or animal models, it was important to assess the relationship between tumors and changes in free amino acids (FAA) profiles of blood or serum of cancer patients. It is worth acknowledging that such a global approach had not been possible without recently developed technology. Cancer cells have unique metabolic requirements^25^, ^26^ and exhibit a deregulated metabolic phenotype. Recent advances in studying metabolomics has helped to gain a comprehensive look at global changes in metabolites, such as FAA and free fatty acids (FFA). However, an in-depth review of metabolic profiles of tumor samples is beyond the scope of this review. Therefore, we will focus on FAA profiling of blood and serum samples of cancer patients.

In line with the experimental observations mentioned above showing that amino acids are important building blocks for tumors, several reports demonstrate that tumors directly influence plasma free amino acid (PFAA) profiles. Threonine, serine, and glycine are significantly reduced in the serum of lung cancer patients. PFAA are tumor type–specific, as there was no decrease in these three FAA in breast cancer patients.^27^ In fact, other groups report similar observations demonstrating that different types of cancer were associated with specific PFAA profiles.^28^, ^29^ Proenza et al described that lung and breast cancer patients exhibit a decrease in blood FAA content, including decreased glutamine, serine, and glycine levels.^30^ The authors suggested that such alterations might be due to increased amino acid demand of cancer cells. Miyagi et al confirmed altered PFAA profiles in lung, gastric, colorectal, breast, and prostate cancer patients.^31^ Interestingly, changes in PFAA were already observed in patients with early-stage tumors. This might indicate that the effects observed are due to a direct relationship between the tumor and the host metabolism rather than a reflection of the patient’s nutritional status. Thus it is tempting to speculate that tumor cells consume and take advantage of specific FAA from the plasma pool.

Taken together, the experimental findings from in vitro and in vivo studies using ^14^C-glycine,^19^, ^20^, ^21^ as well as FAA metabolic profiling from blood and serum samples of cancer patients,^27^, ^28^, ^29^, ^30^, ^31^ provide evidence that protein and FAA metabolism are important features of the cancer macroenvironment. Importantly, changes in amino acid serum profiles might have a potential for early cancer detection.

### Lipid Metabolism

Dysregulated lipid metabolism is a hallmark of cancer. Lipids serve as the structural and functional domains on the scaffold of proteins, as fat depots, and as signaling molecules. Functions of lipids are critical in malignant tumors as they are necessary not only for providing the membrane constituents of proliferating cells but also for energetic, biophysical, and signaling pathways that drive tumorigenesis.^32^ In addition, cancer-specific modifications of the lipid metabolism can affect the production of specific signaling lipids, such as factors derived from poly-unsaturated fatty acids (FA) and alter the availability of specific FA pools required for protein modification. These changes may profoundly affect the tumor macroenvironment.

In 1953 Medes et al found that cancer tissues are able to synthesize lipids de novo, in particular enormous amounts of FA and phospholipids. They also demonstrated that the amount of lipid synthesis in cancer tissue is comparable to that in liver.^33^ Recently, Nieman et al^34^ described that adipocytes sustain human ovarian cancer peritoneal metastases by providing energy for rapid tumor growth. Omental adipocytes promote homing, migration, and invasion of ovarian cancer cells. Co-culture of adipocytes and ovarian cancer cells demonstrated transfer of lipids from adipocytes to cancer cells, enhanced lipolysis in adipocytes, and elevated β-oxidation in cancer cells. Metastasized ovarian cancer cells showed upregulation of fatty acid binding protein 4 (FABP4), especially in the adipocyte–tumor interface and pharmacological inhibition of FABP4 substantially impaired ovarian metastases in mice.^34^

Since the pivotal observation of the important role of fatty acid synthase in cancer cell growth,^35^ numerous studies have confirmed increased de novo lipogenesis in neoplastic tissues. These effects can be reversed through inhibition of enzymes involved in FA biosynthesis pathways.^36^ Various pharmacological inhibitors of fatty acid synthase were shown to be effective in the chemoprevention of breast cancer in HER2/neu transgenic mice. Inhibition of FA desaturation following the ablation of stearoyl-CoA desaturase-1 caused ER stress, cell cycle inhibition, and apoptosis of cancer cells.^37^ ATP-citrate lyase is the rate-limiting cytosolic enzyme responsible for the synthesis of acetyl-CoA in many tissues. It is also an essential regulator in histone acetylation, thereby linking FA metabolism to gene regulation.^38^ Inhibition of ATP-citrate lyase was found to reduce hepatic cholesterol levels and FA synthesis^39^ and to decrease tumor formation in lung and prostate xenografts.^40^ Monoacylglycerol lipase (MAGL) has been shown to be associated with aggressive properties of cancer cells. It hydrolyzes 2-arachidonyl glycerol of the endo-cannabinnoid pathway and other monoacylglycerols. Inhibition of MAGL causes accumulation of monoacylglycerols and reduction of FFA. Overexpression of MAGL in human cancer cell lines increased the aggressive properties of cancer cells, which were reversed by MAGL inhibition. Importantly, human high-grade ovarian cancers are associated with enhanced expression and elevated MAGL activity.^41^

#### Plasma Lipids

De novo lipogenesis is considered to be the primary source of FA available for lipid synthesis in cancer cells. However, cancer cells do not solely rely on de novo lipogenesis but also use exogenous FA for membrane synthesis and for the synthesis of oncogenic signalling lipids such as ceramide-1-phosphate (C1P), platelet-activating factor (PAF), diacylglycerol (DAG), and lysophosphatidic acid (LPA).^42^, ^43^, ^44^ Using an isotopic fatty acid labeling strategy coupled with metabolomic profiling, Louie et al demonstrate that cancer cells also use exogenous fatty acids such as palmitic acid to generate lipids required for proliferation and pro-tumorigenic lipid signaling.^45^

Breast cancer has been shown to be associated with increased plasma FFA concentrations. Linoleic acid does not only induce PAI-1 (a prognostic marker for breast cancer) secretion through SMAD4 (similar to mothers against decapentaplegic-4) but also enhances the migratory potential of the highly invasive MDA-MA-468 breast cancer cell line.^46^ FFA secreted by primary breast cancer into the interstitial fluid were found to inhibit the cytolytic activity of the infiltrating cytotoxic T lymphocytes (CTLs),^47^ providing yet another example of tumor lipid metabolism effecting the tumor micro- and possibly also macroenvironment. A large multicenter study revealed a positive association between serum palmitic acid with a high relative risk of 1.90 for prostate cancer and an inverse association with stearic acid, respectively.^48^ However, despite the increasing evidence for the important role of lipid metabolism in cancer, the mechanisms by which specific lipid species affect incidence and progression of various types of cancer remain elusive.

#### Plasma Lipoproteins

Since lipids play a substantial role in maintaining cellular integrity, it is not surprising that altered lipoprotein patterns also have been associated with malignancies. Patients suffering from various types of hematological neoplasia exhibit significantly lower plasma cholesterol, high-density lipoprotein-cholesterol (HDL-C), and low-density lipoprotein-cholesterol (LDL-C) levels and higher triglyceride (TG) concentrations than body mass index (BMI)-matched healthy controls.^49^ A similar reduction in total cholesterol (TC), HDL-C, and very-low-density lipoprotein-cholesterol (VLDL-C) was observed in patients with oral^50^ and head and neck cancers.^51^ The role of plasma lipids in breast cancer is a subject of controversy. Plasma TC and LDL-C were found to be elevated in breast cancer patients^52^ and were associated with tumor progression,^53^ whereas other studies showed increased TG and VLDL-C but reduced TC, HDL-C, and LDL-C in patients with advanced compared to early-stage breast cancer.^54^ In patients with metastatic disease, a similar reduction of TC, LDL-C, HDL-C, and BMI was observed in comparison to patients with non-metastatic tumors. However, serum TG was also decreased in these patients.^55^

In general, low plasma LDL-C levels are robustly correlated with cancer. Surprisingly, however, genetically decreased LDL-C in patients with three polymorphic genotypes—proprotein convertase subtilisin/kexin (PCSK) type 9, ATP-binding cassette sub-family G (ABCG) member 8, and apolipoprotein (APO)E—was not seen.^56^ In addition, meta-analyses of randomized controlled trials of cholesterol reduction found no significant rise in cancer mortality.^57^ It seems, therefore, that low LDL-C levels per se do not cause cancer. It is conceivable that low LDL-C levels might be caused by tumor effects on the macroenvironment.

## Effects of Cancer on The Macroenvironment: Cancer-Associated Cachexia

Cancer-associated cachexia (CAC) is a multi-factorial syndrome characterized by progressive loss of skeletal muscle mass with or without loss of fat mass that cannot be reversed by conventional nutritional support.^58^ CAC is characterized by anorexia, anemia, lipolysis, and insulin resistance. It is estimated that 15%–20% of deaths of cancer patients can be attributed to cachexia. The highest prevalence is seen in patients suffering from gastrointestinal and pancreatic adenocarcinoma with 80%–90% incidence followed by prostate and lung cancer.^59^

Clinically, cachexia should be suspected if an involuntary weight loss of>5% of the premorbid weight occurs within a 6-month period. While anorexia also may occur concomitantly, the drop in caloric intake alone does not explain the body composition changes seen in cachexia. Moreover, cachexia may progress even in the absence of anorexia.^60^ The major influence of the tumor on the macroenvironment appears to be related to excess of cytokines in the serum: (1) many tumors secrete pro-inflammatory factors (eg, tumor necrosis factor alpha [TNFα], IL-6) and pro-catabolic factors (eg, zinc α_2_-glycoprotein [ZAG]); and (2) factors released by the host as a response (eg, interferon gamma [IFNγ] and ZAG),^61^ which are responsible for promoting degradative pathways in skeletal muscle and adipose tissue. In the following sections we delineate the effects a cachexia-inducing tumor exerts on the host via several mechanisms.

### Systemic Inflammation

Many lines of investigations prove beyond a reasonable doubt that a multifactorial in situ network of inflammation governs various intricate signaling processes that advance tumor development and progression. In addition to microenvironmental effects, inflammatory responses in the macroenvironment are associated with increased levels of inflammatory mediators (eg, IL-6, TNFα, IL-1, and IFNγ)^62^ and acute-phase proteins that lead to hypermetabolism and weight loss in patients with CAC.^63^ Based on these findings, many studies attempted to define potential diagnostic markers for CAC. It has been shown that in advanced stages of cancer, IL-1β is more strongly associated than other cytokines with clinical features of cachexia such as general weakness, loss of appetite, weight loss, and sarcopenia.^64^ Interestingly, despite the high levels of plasma TNFα and IL-6 in patients with non-small cell lung cancer compared with healthy volunteers, the difference in plasma TNFα and IL-6 between cachectic and non-cachectic patients is not significant.^65^ It seems possible that a set of cytokines has to work in concert to induce CAC and that a single factor might therefore be poorly predictive of CAC.

The mechanistic interaction between systemic inflammation and tumor development in patients has not yet been fully elucidated. There is, however, increasing experimental evidence for a causal relationship between systemic inflammation and features of CAC. In experimental CAC models, administration of many of the cytokines listed above led to anorexia, weight loss, acute-phase protein response, protein and fat breakdown, and increased levels of cortisol and glucagon, as well as decreased insulin levels, insulin resistance, anemia, fever, and elevated energy expenditure.^66^ Increased levels of IL-6 in a murine colon carcinoma model correlated with the development of cachexia, whereas treatment with monoclonal antibody to murine IL-6 suppressed it.^67^ Similarly, neutralizing endogenous TNFα/cachectin production with antibodies reduced tissue wasting and tumor weights of methylcholanthrene-induced sarcoma (MCG-101), as well as Lewis lung carcinoma.^68^

### Adipose Tissue Depletion and Hypermetabolism

Loss of adipose tissue is one of the hallmarks of CAC. A remarkable decrease in size of adipocytes was observed in cachectic mice^69^ and patients.^70^ TG depletion in adipose tissue is caused by aberrant production of several factors derived from tumors and/or host tissues.^61^ These factors include inflammatory cytokines such as TNFα and pro-lipolytic factors such as lipid-mobilizing factor and ZAG, which have a direct lipolytic effect and also sensitize adipocytes to lipolytic stimuli.^71^ Both lipid-mobilizing factor and ZAG induce lipolysis through the canonical adenylyl cyclase-cAMP–mediated mechanism and subsequent activation of hormone-sensitive lipase (HSL).^61^ Remarkably, elevated levels of ZAG, as well as TNFα and IL-6, did not induce depletion of adipose tissue in Lewis lung carcinoma–bearing mice lacking adipose tissue triglyceride lipase (ATGL), pointing to a central role of ATGL in the pathogenesis of CAC.^72^ In addition to lipolysis as the most predominant cause, decreased lipogenesis and FA uptake could partially explain TG depletion. Essential transcript factors (eg, C/EBP, SREBP^69^) and lipogenesis enzymes (eg, fatty acid synthase, citrate cleavage enzyme^73^) are associated with tumor progression in mouse cachexia models.

Adipose tissue is a potent source of energy, constituting about 90% of adult fuel reserves. Instead of being viewed as a passive calorie reservoir, it is now recognized as a highly active metabolic as well as endocrine organ profoundly impacting on the host energy metabolism via adipokines.^74^ It is well established that loss of adipose tissue results in extensive fatty acid and glycerol mobilization and circulation in cachectic patients due to increased lipolysis compared with patients with non-cachectic cancer or healthy subjects.^75^, ^76^ Increased oxidation of fat and glucose along with elevated energy expenditure is frequently observed in a wide spectrum of different cancers,^75^ whereas impaired capacity to oxidize lipids also was found in weight-losing gastrointestinal cancer patients.^76^ Increased energy expenditure also could arise from tumor-derived factors irrespective of their pro-lipolytic activity. For example, injection of lipid-mobilizing factor from cachectic cancer patients promotes whole body fatty acid oxidation in mice.^77^ Tumors are metabolically active and since they have the potential to adapt rapidly they might even take advantage of metabolic changes. Considering the potential use of lipoproteins by tumors in cancer patients and in experimental models,^78^ it is conceivable that the increased flux of lipids into circulation due to loss of adipose depots is not entirely wasted in the “hyper-metabolic sink” but might in part be used by the tumor itself.

### Muscle Atrophy

Cachexia-related muscle wasting results from a disturbance of the tightly regulated balance of muscle protein breakdown and synthesis.^79^ Intracellular protein degradation involved in cachexia can be mediated by three processes: the lysosomal mechanism, a Ca^2+^-dependent mechanism, and the ATP-ubiquitin–dependent proteolytic pathway (UPP). The latter is considered to be preferentially activated.^80^ Several mechanisms may trigger the ATP-ubiquitin–dependent proteolytic pathway such as a set of cytokines found in CAC (eg, TNFα, IL-1, IL-6, and IFNγ). NFκB, a central mediator downstream of various pro-inflammatory factors, regulates muscle protein degradation and expression of the ubiquitin-proteasome proteolytic pathway in response to proteolysis-inducing factor (PIF).^81^ Muscle STAT3 activation by IL-6 is a common feature of cancer-associated muscle wasting.^82^ Inhibition of IL-6/JAK/STAT3 reduced muscle atrophy in cancer, indicating that IL-6/STAT3 is a critical mediator axis of muscle wasting in cancer cachexia induced by high levels of IL-6.^82^

Cytokines such as those mentioned above induce systemic inflammation. Indeed, insulin resistance and sensitivity to systemic inflammation were observed in patients with various types of tumors and were associated with CAC.^83^ There also is evidence that cachexia-associated insulin resistance could result in increased protein degradation of skeletal muscle.^83^ Increased energy expenditure in cachectic cancer patients suffering from gastrointestinal adenocarcinoma might, at least in part, be related to increased expression of uncoupling protein-3 in muscle, which may contribute to tissue catabolism.^84^

Besides protein breakdown, a reduction in the rate of muscle protein synthesis in weight-losing cancer patients also has been described in cancer patients. In some cases, muscle protein synthesis decreased dramatically compared with healthy controls, whereas whole body rates of protein synthesis and degradation do not differ significantly.^85^

## Systemic Metabolic Diseases With Possible Influence on Tumor Development

### Obesity

Human obesity is a complex disease resulting from a combination of elevated caloric intake and a relative lack of physical activity. Hippocrates was the first one to note the relation between obesity and reduced life expectancy. In one of his medical works he stated that “Sudden death is more common in those who are naturally fat than in the lean”. Various studies have provided ample evidence that obesity is risk factor linked with chronic illnesses, and is not only restricted to diabetes, heart diseases, dyslipidemia, inflammatory diseases, and hypertension. In 2003, a landmark study was performed by the American Cancer Society analyzing the influence of excess body weight on the risk of cancer-related deaths in a large population of 900,000 American adults. The prospective investigation showed that men and women with a BMI of at least 40.0 had a death rate from all cancers combined of 52%, which was 88% higher than their normal-weight counterparts.^86^ Additional studies demonstrate an increased risk for various cancer types such as colon and renal cancers, leukemia, non-Hodgkin lymphoma, and esophageal adenocarcinoma in both sexes; endometrial, ovarian, gallbladder, breast, and pancreas carcinomas in women; colon, breast, and endometrial cancers in postmenopausal women^87^; and malignant melanoma, and stomach, prostate, and rectal cancers in men.^88^

The basic mechanism(s) linking obesity to tumor-initiating events remain largely elusive. Two main mechanistic connections have been suggested that may causally link obesity and increased fat mass with cancer progression: (1) altered signaling events, and (2) changes in the local and systemic levels of adipocyte-derived factors. This altered physiological state may induce an enhanced mitogeneic effect shaping the tumor microenvironment through autocrine and paracrine signaling combined with infiltration of immune cells and inflammation.^89^ Adipose tissue secretes various polypeptide hormones, adipokines, leptin, and plasminogen activation inhibior-1 (PAI-1), which have been reported to be involved in cancer development and progression.^90^ Cancer progression could be induced by the activation of PI3K, MAPK, and STAT3 pathways, respectively.^91^ Excess adipose tissue in obesity is associated with higher levels of pro-inflammatory cytokines, including TNFα, IL-2, IL-6, IL-8, IL-10, PGE2, and monocyte chemoattractant protein-1 (MCP-1). Activation of NFκB also may play a major role through various inflammatory mechanisms.^92^ Though there are several mechanisms proposed to be involved in obesity-associated cancer, the exact molecular events remain unclear.

### Diabetes

Epidemiological data suggest that there is an association between the incidence of a wide variety of malignancies and diabetes. A causative relationship has not been proven so far, but biological mechanisms that support this theory have been found. However, it has to be kept in mind that diabetes and malignant tumors have common risk factors.^93^ A large number of cohort and case-control studies, as well as meta-analyses of these studies, support the evidence that the incidences of many different cancers are increased in diabetic patients.^94^

A meta-analysis of case-control and cohort studies indicated an association of diabetes mellitus with an increased risk for colon cancer in both men and women, whereas rectal cancer showed this association only in male patients. Analysis of the seven studies that controlled for known confounders such as smoking or obesity still showed this association, which was also independent of physical activity.^95^ A meta-analysis of 21 studies, including case-control and cohort studies, demonstrated a statistically significant association between diabetes and colorectal cancer incidence without heterogeneity between the different studies. In this analysis the risks for colon cancer and rectal cancer were similar. Even the analysis of studies correcting for the well-known confounders, physical activity and BMI resulted in a positive association between diabetes and colorectal cancer.^96^ In addition, a meta-analysis including more than 3 million patients showed that diabetic patients had a significantly higher risk of colorectal cancer; even when only studies controlling for BMI and smoking were included, an association between diabetes and risk of colorectal cancer was found.^97^

In a meta-analysis of 36 studies stratified by study design, diabetes mellitus was associated with a higher incidence of bladder cancer in case-control and cohort studies with an even higher risk within the first 5 years.^98^ An association between breast cancer and diabetes was demonstrated in a meta-analysis of 20 studies with a significantly increased risk of 20% of diabetic women developing breast cancer. In an additional analysis stratified for menopausal status, diabetes was shown to be associated with breast cancer only in postmenopausal women.^96^ However, type I diabetes and diabetes in premenopausal patients did not exhibit an increased risk of breast cancer.^99^

The association of diabetes mellitus and malignancies of the gastrointestinal tract also has been investigated in a large number of studies. In a cohort study of 929 diabetic patients and 1,126 controls, a 2.75-fold increase of gastrointestinal malignancies, including gastric, hepatic, colon, and pancreatic cancers, was demonstrated.^100^ A meta-analysis of 30 studies showed an increased risk for pancreatic cancer in diabetic patients, especially for those with a history of diabetes of less than 5 years duration^101^; this also was confirmed in another meta-analysis of 20 studies.^102^ An increased risk of gallbladder cancer and extrahepatic cholangiocarcinoma was found in a meta-analysis of 21 studies including eight case-control and 13 prospective cohort studies. Studies controlling for the two most important confounders of biliary tract cancer showed an increased, but not statistically significant association, of diabetes mellitus with biliary tract cancer.^103^ In a meta-analysis of 18 cohort studies, 13 studies showed an increased risk of hepatocellular cancer in diabetic patients. This positive association was even found when only studies controlled for the most important confounding factors, including hepatitis B and C infection or alcohol consumption.^104^ A meta-analysis of 16 studies showed that the risk of endometrial cancer was increased in diabetic patients, with a stronger association in the case-control studies in comparison to cohort studies.^105^ In a prospective cohort study of 36,773 women, a diabetic condition was associated with a twofold increased risk even when adjusted for confounders like age, MI, and total physical activity ^106^.

The mechanisms leading to this increased risk of malignant tumors in the diabetic population have been investigated and a number of genetic pathways have been implicated in this process. Hyperglycemia itself, however, also interacts with tumor cells. High glucose levels have a direct effect on cancer cells leading to increased proliferation, inducing mutations of various genes, augmenting invasion and migration, and resetting signaling pathways in tumor cells.^107^ Hyperglycemia, hyperinsulinemia, and chronic inflammation have been discussed as mechanisms by which diabetes might promote growth of malignant tumors.^108^ The dependence of malignant cells on glycolysis has been described as the Warburg hypothesis.^108^, ^109^ On the other hand, cell culture results indicate that the glucose transporter GLUT1 is upregulated and that cells have an enhanced glucose uptake even in a low glucose environment.^110^ Transcriptional profiling of a cell line model of transformation showed a significant correlation of 54 genes between cancer and metabolic conditions. In the same model, 11 of 13 medications for treatment of metabolic disease suppressed colony formation; however, they did not affect cellular growth.^111^

In contrast to the large number of studies described above, diabetes appears to have an opposite effect on the pathogenesis of prostate cancer. In a prospective cohort study, a diabetic metabolic state was associated with a risk reduction of 25% of prostate cancer.^93^ The risk for developing prostate cancer declined briefly after the onset of diabetes mellitus and this reduction continued for the following 15 years. This declining risk might be caused by a drop in testosterone levels.^112^ Considering the ample evidence associating diabetes with cancer, it is clear that more investigations are needed to clarify the mechanism by which diabetes can cause or, in some circumstances, even prevent cancer.

## Clinical Implications of The Tumor Macroenviroment

### Macroenvironment and Impact on Patient’s Clinical Outcome

Until now, cancer-staging systems and prognostic stratification tools for patients exclusively rely on tumor-related clinical or histopathological factors. Tumor size, number, and location of metastatic lesions, tumor grading, or other histomorphological features like vascular invasion or tumor necrosis provide the basis for individual risk assessment in daily clinical routine.^113^ However, in addition to novel molecular markers and multi-gene assays, the simple observation that patients with nearly identical tumor burden show different clinical signs, including thromboembolic events, fever, or tumor cachexia, suggests that the interaction between the tumor and its macroenvironment influences life quality and survival of cancer patients. In this context, the systemic inflammation that is determined by production and systemic secretion of soluble factors of the tumor cells and the tumor microenvironment has been previously reported as a potentially useful indicator of the patient’s clinical outcome. More than 10 years ago, the first study reported that an elevated C-reactive protein level, a commonly used surrogate marker indicating the degree of systemic inflammatory response, is predictive for the duration of cancer-specific and non-cancer survival in patients suffering from colorectal, gastric, breast, or lung cancer.^114^ A long list of other studies confirmed these findings in different cancer entities and under different clinical scenarios, which established the systemic inflammatory response as a potentially prognostic indicator in cancer patients.^115^, ^116^ In addition to the originally used C-reactive protein, a series of other blood-based markers or combinations have been proposed as possible indicators of the systemic inflammatory response. These include the modified Glasgow prognosis score, a combination of albumin and C-reactive protein levels, which divides patients into different risk groups.^117^ Other useful markers indicative for the systemic inflammatory response include the neutrophil to lymphocyte ratio,^118^ the lymphocyte to monocyte ratio,^119^ and other plasma proteins like fibrinogen levels.^120^ As already mentioned above, the systemic inflammatory response is also strongly and causally linked to CAC. Taken together, several lines of evidence support the theory that the systemic inflammatory response impacts the clinical course of cancer patients. Therefore, integrating blood-based surrogate markers into established clinical staging systems might improve the predictive ability of currently used prognostic risk assessment tools.

### Solid Pediatric Tumors—A Special Case?

Basic principles of adulthood cancer do not necessarily apply to solid pediatric tumors such as hepatoblastoma (HB), the most common liver tumor in infancy, or neuroblastoma (NB), the most common extracranial solid tumor in children. These entities are of embryological origin, and have distinct genetic alterations, unique growth patterns, and specific prognoses. NB may have different genetic clones within one individual lesion and either may progress to a chemotherapy-resistant malignancy or mature to a “benign” ganglioneuroma. Consequently, it must be assumed that environmental factors influencing each specific lesion could be distinctly different from adulthood cancer. This section elucidates current knowledge about the macro- and microenvironment in children with solid pediatric tumors and focuses on major principles and potential therapeutic strategies for pediatric oncology in the future.

#### The Metabolic Environment

As early as 1930 Warburg described the dependence of tumors cells on glycolysis even in the presence of adequate oxygen supply (“aerobic glycolysis”). Today we understand that this disproportional metabolism of glucose into lactate^121^ is mediated by upregulation of glycolysis in the cytosol and downregulation of glucose oxidation by the mitochondria.^122^ Molecular abnormalities of glucose metabolism have been investigated in solid pediatric tumors. Park et al^123^ showed that hypoglycemia increased induction of VEGF expression via the protein kinase C pathway in human hepatoblastoma cells. It is well known that VEGF plays a central role in angiogenesis and that VEGF expression can be influenced by a variety of environmental stresses such as nutrient deprivation and hypoxia. Terashima et al^124^ supported this finding of increased VEGF expression under glucose deprivation in HepG2 cells. Like adulthood cancers, this pediatric tumor obviously can initiate molecular strategies to escape metabolic deprivation.

Using neuroblastoma cells (SH-SY5Y and SK-N-BE) Navratilova et al demonstrated that tetrathiomolybdate (TMD), a drug that exhibits anti-angiogenic and tumor-suppressing effects increased glucose uptake, production of lactate, and activation of Akt and AMPK signaling pathways as angiogenic “escape strategies” of NB cells under low glucose conditions.^125^ Under low glucose conditions, these effects lead to a significant decrease of intracellular ATP supply and apoptosis. The authors concluded that TMD in combination with dietary restrictions could be a suitable agent for the treatment of NB.

CAC represents a hypercatabolic syndrome characterized by depletion of adipose and protein tissues.^72^, ^126^ Recent studies in adulthood cancer unraveled novel mediators with the potential for pharmacological inhibition.^127^ In childhood oncology, severe CAC does not seem to be a major clinical problem. Thus in the literature there are almost no reports about the energy homeostasis in solid pediatric tumors. It remains rather unclear which molecular strategies these unique embryologic tumors employ to harvest energy. Nevertheless, such studies could reveal subclinical interactions with the host’s energy homeostasis or uncover distinct metabolic pathways for each tumor entity.

#### The Immunologic Environment

Tumor cells can manage to escape the anti-tumor immune responses. Revealing the underlying mechanisms for solid pediatric tumors could foster development of tumor-specific and immunologic anti-cancer therapies. The tumor microenvironment certainly plays a central role in this context as it presents the “stage” for the interaction between proliferating tumor cells, tumor stroma on the one hand and blood vessels supplying soluble anti-cancer factors or inflammatory cells on the other hand.

For NB, Pistoia et al^128^ recently described several immune escape mechanisms. These include (1) an impaired expression of HLA class I antigens leading to a defective antigen presentation and immune response by the host, (2) expression of several immunosuppressive molecules, and (3) recruitment of immunosuppressive cells impairing anti-tumor immune responses. Such immunological escape mechanisms could be treated pharmacologically.^129^ Immunotherapy with lenalidomide enhanced activation of natural killer cells and inhibited their suppression by NB induced IL-6 or transforming growth factor-ß1 within the tumor environment.

#### The Metastatic Environment

The fate of tumor cells reaching distant organs depends on local factors within the “new” microenvironment. Such tissue-derived factors can influence the viability, proliferation, cell adhesion and motility, chemotaxis, or apoptosis.^130^ In children with high-risk NB, pulmonary metastases are crucial for the long-term outcome. In an orthotopic mouse model for human neuroblastoma metastases (Mhh-NB11 and SH-SY5Y), Maman et al^131^ showed that lung-derived factors significantly reduced the viability of micro-NB cells by upregulating the expression of pro-apoptotic genes, inducing cell cycle arrest and decreasing ERK and FAK phosphorylation. The authors concluded that further insights into distant organ environment could reveal therapeutic options against NB metastases. In conclusion, various metabolic and immunologic factors of the macro- and microenvironment within the tumor or distant organs seem to play an essential role for the morbidity and mortality of children with NB and HB.

### Targeting the Tumor Macroenvironment

In the previous sections we delineated various tumor-induced effects on the macroenvironment, such as tumor-induced systemic inflammation, that potentially modulate metabolism and induce cachexia. Therapeutic efforts to block the actions of, for example, macrophage-secreted substances, may slow the progression of tumor effects on the macroenvironment such as cachexia. Anti-inflammatory compounds, such as cyclo-oxygenase 2 inhibitors, appear to be efficacious in the reduction of cachexia in animals,^132^ as well as in patients.^133^ Resveratrol, an inhibitor of NFκB activation, can inhibit muscle protein degradation in experimental CAC.^134^ Genetic ablation of IL-6 in mice has been shown to suppress both tumor growth and weight loss in an experimental cachexia model, implying that host-derived cytokines also could be considered as therapeutic targets.^135^ Targeting the tumor macroenviroment in patients suffering from cachexia through anti-inflammatory therapy not only may ameliorate the physical condition of patients but might also disrupt the feedback of the macroenvrionment to the tumors thus providing novel therapeutic targets.

Dietary modification such as caloric restriction has been shown to decrease tumor initiation and progression in model systems of cancer. In breast tumor-bearing mice, it induced metabolic and signaling changes that affect stroma and tumor cells, resulting in reduction of tumor proliferation and consequent metastases.^136^ In murine models of triple-negative breast cancer, a 30% reduction in daily total caloric intake provided significant tumor regression compared to alternate-day feeding, and greater regression when combining radiation and dietary modification.^137^ However, despite several efforts, no solid evidence exists to substantiate that caloric restriction or other dietary interventions can reduce tumor growth in cancer patients.

HMG-CoA (3-hydroxy-3-methylglutaryl-coenzyme A) reductase is the rate-limiting enzyme in the biosynthesis of isoprenoid compounds, including cholesterol, dolichol, and ubiquinone.^138^ Its inhibitors, statins, have been used to treat hypercholesterolemia but also display anti-tumor effects against various types of cancer in tumor models.^139^ Anti-tumor properties of statins have not been fully elucidated but might be attributed to (1) blocking of the de novo cholesterol synthesis, which is crucial in the maintenance of cellular membrane and integrity; (2) impeding the transition of G1-S in the cell cycle; (3) interference with cell signaling (eg, Ras and Rho family GTPases dependent on isoprenoids for membrane anchoring^140^); and (4) apoptosis induction through depletion of geranylgeranylated proteins ^141^ or deregulation of pro-apoptotic BAX and anti-apoptotic BCL-2 expression.^142^ Intracellular cholesterol levels are tightly regulated by a homeostasis network, including LDL uptake, which could compensate for a high cholesterol demand while cellular cholesterol supplied from de novo synthesis is insufficient. In fact, the importance of the LDL receptor in tumorigenesis is generating increasing interest.^143^ Thus, reduction of circulating lipids might reduce nutrient supply to the tumor and thereby lead to tumor suppression. In fact, we recently were able to show that the lipid-lowering drug fenofibrate suppresses B-cell lymphoma growth via a systemic mechanism.^78^

## Conclusion

This review is intended to provide convincing arguments for the tumor macroenvironment concept since we believe it to be very useful to explore the effects tumors exert on the entire complex organism. The multiple interfaces between tumor cells, tumor stroma, including vasculature and immune cells, and the surrounding tissue and organs are a fascinating environment to study the interplay of the various components. This will help to understand the biology and the properties of malignant tumors much better and will undoubtedly support the establishment of new therapy and prevention concepts.
