# Corrigendum to “Tumor Macroenvironment and Metabolism” [Seminars in Oncology, Vol 41, No 2, April 2014, pp 281–295]

**DOI:** 10.1053/j.seminoncol.2014.07.005

**Published:** 2014-08

**Authors:** Wael Al-Zoughbi, Jianfeng Huang, Ganapathy S. Paramasivan, Holger Till, Martin Pichler, Barbara Guertl-Lackner, Gerald Hoefler

**Affiliations:** a Institute of Pathology, Medical University of Graz, Graz, Austria; b Department of Paediatric and Adolescent Surgery, Medical University of Graz, Graz, Austria; c Department of Internal Medicine, Medical University of Graz, Graz, Austria

The authors regret that the first author’s name was misspelled in the printed publication.

